# Advances in Starch Nanoparticle for Emulsion Stabilization

**DOI:** 10.3390/foods12122425

**Published:** 2023-06-20

**Authors:** Jianwei Zhou, Meimei Guo, Yu Qin, Wenjun Wang, Ruiling Lv, Enbo Xu, Tian Ding, Donghong Liu, Zhengzong Wu

**Affiliations:** 1 School of Mechanical and Energy Engineering, NingboTech University, Ningbo 315100, China; hizjw@163.com (J.Z.); 22113075@zju.edu.cn (M.G.); qinyuu10@163.com (Y.Q.); lvruiling@zju.edu.cn (R.L.); 2 Ningbo Innovation Center, Zhejiang University, Ningbo 315100, China; dhliu@zju.edu.cn; 3 Innovation Center of Yangtze River Delta, Zhejiang University, Jiaxing 314102, China; wangwj@zju.edu.cn (W.W.); enboxu@zju.edu.cn (E.X.); tding@zju.edu.cn (T.D.); 4 State Key Laboratory of Fluid Power and Mechatronic Systems, National Engineering Laboratory of Intelligent Food Technology and Equipment, Zhejiang Key Laboratory for Agro-Food Processing, Fuli Institute of Food Science, College of Biosystems Engineering and Food Science, Zhejiang University, Hangzhou 310058, China; 5 Food Laboratory of Zhongyuan, Luohe 462044, China; 6 State Key Laboratory of Biobased Material and Green Papermaking, School of Food Science and Engineering, Qilu University of Technology, Shandong Academy of Sciences, Jinan 250353, China

**Keywords:** starch nanoparticle, microstructure, preparation strategy, stability, Pickering emulsion

## Abstract

Starch nanoparticles (SNPs) are generally defined as starch grains smaller than 600–1000 nm produced from a series of physical, chemical, or biologically modified starches. Many studies have reported the preparation and modification of SNPs, which are mostly based on the traditional “top-down” strategy. The preparation process generally has problems with process complexity, long reaction periods, low yield, high energy consumption, poor repeatability, etc. A “bottom-up” strategy, such as an anti-solvent method, is proven to be suitable for the preparation of SNPs, and they are synthesized with small particle size, good repeatability, a low requirement on equipment, simple operation, and great development potential. The surface of raw starch contains a large amount of hydroxyl and has a high degree of hydrophilicity, while SNP is a potential emulsifier for food and non-food applications.

## 1. Introduction

Starch is a natural biological polymer, which is synthesized by plant photosynthesis and a series of complex biochemical reactions, widely present in various types of plant crops, and it is the second-largest biomass in nature after cellulose [1]. Starch is the most important energy storage matrix for plants on the planet, and the core source of carbohydrates in the human diet plays an irreplaceable role in living organisms. As a semi-crystalline biopolymer, the structure of starch is complex, and can be divided into multiple levels, such as molecular, crystalline, and particle structure [2]. A starch nanoparticle is generally defined as starch grains smaller than 1000 nm produced by a series of physical, chemical, or biologically modified starches [3]. It can be divided into starch nanocrystals (SNCs) and starch nanoparticles (SNPs) according to differences in crystal structure caused by preparation methods [4]. Compared with the raw starch, the starch nanoparticle has a unique nano-effect due to its submicron-scale size and higher specific surface area. They are embodied in weak light scattering, high adsorption capacity, excellent solubility, and bioaccessibility [5,6]. In recent years, under the global environment of sustainable development, the starch nanoparticle has been widely concerned by researchers because of its green environmental protection, being non-allergenic, a and rich source of raw materials. The physicochemical properties of starch nanoparticles are affected by starch from different plant sources, preparation methods, and application scenarios. Therefore, the production process of starch nanoparticles can be optimized for the functional characteristics required for different purposes in the food industry.

Pickering emulsions refer to emulsions stabilized by ultrafine solid particles [7]. Studies have shown that modified micron-sized and nano-sized solid particles can form physical barriers with certain mechanical strength through directional adsorption at the oil-water interface to improve the stability of the emulsion [8]. Compared with conventional surfactants, once solid particles are successfully adsorbed at the oil-water interface, a higher desorption energy needs to be introduced to separate them from the two-phase interface, and the desorption energy is affected by particle size, oil-water interface tension, water-oil-particle three-phase contact angle, etc. [9]. The emulsions become shear-thinning at high oil concentrations and the consistency index of emulsion increases with the increase in nanoparticle and oil.

Concentrations [10]. Therefore, the thermodynamically irreversible adsorption of Pickering emulsion at the oil-water interface allows for good stability to resist coalescence and Ostwald Ripening [11]. However, the interfacial tension between mineral oil and water decreases with time at different concentrations of nanoparticles [12]. Based on the concept of green, healthy, and sustainable development, the research topic of Pickering emulsifiers has gradually shifted from inorganic particles such as titanium dioxide, silica, and clay to food-grade colloidal particles such as carbohydrates, proteins, and lipids [13]. The starch nanoparticle is a high-quality raw material for the preparation of Pickering emulsifier because it has biodegradable, biocompatible, low-cost, and other advantages [14].

Starch-based Pickering emulsion, as a degradable and nontoxic carrier, has become a hot topic. It is possible that the modified starch based on Pickering emulsion could be presented as promising culture substrates for various biomedical applications due to its lack of significant detrimental effect on cell adhesion [15]. Nanoparticle stabilized emulsions with starch can improve the stability of emulsion in beverages as compared to stabilized surfactant and biopolymer emulsions [16]. Pickering emulsion stabilized by taro SNPs can load tea polyphenols with a retention rate of up to 67% [17]. The biodegradability, excellent mechanical properties, and low permeability to the vapor of nano starch make it a potential to be an ideal reinforcing material for the preparation of starch-based nanocomposite membranes [18]. Starch nanoparticles can be used as adsorbents to remove aromatic and organic pollutants after chemical modification [19].

Therefore, this paper reviews the preparation methods of starch nanoparticles and the stability mechanism of Pickering emulsion, and introduces the application of starch nanoparticles in emulsion.

## 2. Multiscale Structure of Starch and Starch Nanoparticle

### 2.1. Molecular Chain, Crystal, and Granule Structure of Starch

Chemically, starch is polymerized by D-glucose residues linked by α-1,4-glycoside bonds and α-1,6-glycoside bonds. According to the different links, the starch molecules can be divided into amylose and amylopectin. The former is a linear polysaccharide formed by the α-1,4-glycoside bond and a small amount (<1%) α-1,6-glycosidic bond polymerization, and the latter is a large number of side chains linked by α-1,4-glycoside bonds through 5% to 6% of the α-1,6-glycoside bond to the main chain to form a branched polysaccharide [20]. This is also the basic structure for the formation of starch nanoparticles.

The amylopectin content of most proto-starches is as high as 70~80%, so the amylopectin molecular model is significant for the molecular structure levels of starch. It is suggested that amylopectin molecules can be divided into A-chain (side chain without lateral branches), B-chain (inner chain containing lateral branches), and C-chain (main chain). The chain structure is based on the B-chain as a medium, respectively, linked to the inner A-chain and outer B-chain through α-1,6-glycosidic bonds to form a cluster structure. An amylopectin molecule has only one C-chain and one reducing end. The A-chain and B-chain form double helices in their respective extending directions.

**Figure 1:**
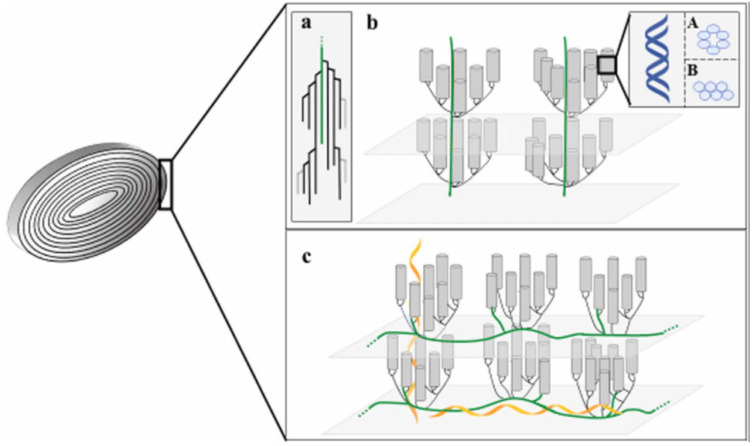
Schematic diagram of starch chain model (**a**), cluster model (**b**), and building block backbone model (**c**) [24]. Reproduced with permission from Apriyanto, A.; Compart, J.; Fettke, J. A review of starch, a unique biopolymer—Structure, metabolism and in planta modifications; published by Elsevier, 2022.

Recently, there are two theories for describing the arrangement of amylopectin C-chains. As shown in Figure 1, one is the cluster model proposed by French [21] and Robin P [22], which holds that the double helical structure of the A and B chains is consistent with the extension direction of the C-chain. Another theory (the building block backbone model) describes that the C-chain is mainly located in the amorphous region, its extending direction perpendicular to the double helical structure formed by the mutual entanglement between the A-chain and the B-chain [23]. On this basis, both theories agree that the central axis of the A-chain and B-chain double helical structures is perpendicular to the surface of starch particles [24]. However, there is no clear conclusion on the distribution of the spiral structure of the amylose molecule inside the starch particles and the winding direction relating to amylopectin. The growth and metabolism of amylose in plants and the role of amylose in starch structure also need further study [25].

**Figure 2:**
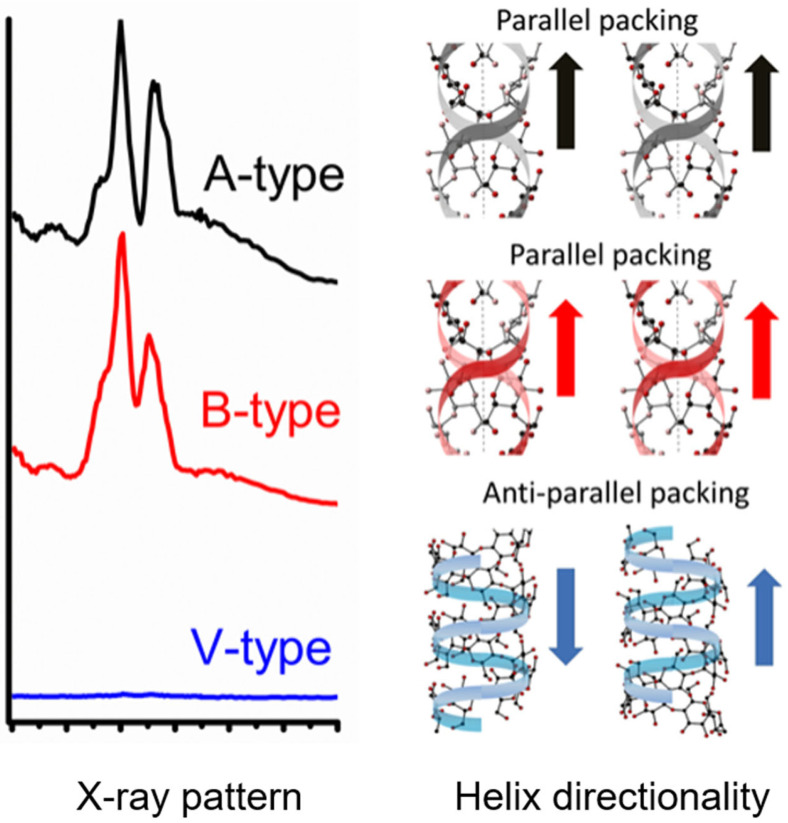
X-ray diffraction patterns and helical structures of A, B, and V type [27]. Reproduced with permission from Kong, L.Y.; Lee, C.; Kim, S.H.; Ziegler, G.R. Characterization of starch polymorphic structures using vibrational sum frequency generation spectroscopy; published by American Chemical Society, 2014.

The crystal form of starch can be determined by the X-ray diffraction pattern. As shown in Figure 2, from the molecular structural domain, amylopectin molecules, and amylose molecules can be entangled with each other by hydrogen bonds in different proportions. Resulting in complex spiral structures, and then assembling them to form crystalline lamellae and amorphous regions. According to the different arrangements of starch molecular chains and water molecules in the crystal domain, starch crystal forms can be divided into A-type and B-type. Type A shows the monoclinic cell configuration of the left-hand double helix of the parallel chain, the water molecules are surrounded by tight and tangent double helixes. The B-type double helix shows the hexagonal cell configuration, which is loosely arranged and contains more water molecules [26].

In addition, the introduction of hydrophobic guest molecules induces a single V-shaped helical structure between starch molecules and guest molecules. Based on the size of the guest molecule, its interaction with the glucose unit will directly determine the form of the hydrophobic cavity that embeds the starch molecule, and then form V-6, V-7, and V-8 helical structures composed of 6, 7, and 8 glucosyl units in a spiral cycle [28].

In recent years, the dominant model is the multi-level starch structure. According to this theory, starch particles are formed by onion-like growth rings with a thickness of 120–500 nm growing from inside to outside. The growth rings are composed of structural units (Blocklets). According to the arrangement density of Blocklets, growth rings are divided into semi-crystalline growth rings and amorphous growth rings, which are alternately arranged similar to a shell [29]. Based on the spiral structure of amylose and amylopectin molecules, the inner blocklets were further divided into crystalline flakes and amorphous flakes, which were arranged alternately. Studies suggest that the growth ring of starch is stacked from Blocklets of size 20–250 nm, with one amylopectin molecule forming one Blocklet and amylose penetrating through it [30,31].

### 2.2. Starch Nanoparticle

#### 2.2.1. Preparation Methods of SNP

According to the different production paths, the preparation methods of the SNP can be divided into “top-down” and “bottom-up”. Where “top-down” refers to the technology of gradually pulverizing and deagglomerating raw starch into nano-sized particles from macroscopic bulky aggregates by a series of physical and chemical means. The traditional method of preparing nano-sized starch mainly carries out micro-structure level scale deagglomerating on large particles of raw starch by physical and chemical methods such as acid hydrolysis, high-pressure homogenization, reaction extrusion, and grinding, thereby generating submicron and nano-sized SNPs [32]. Based on the different preparation principles, the “top-down” method can be further subdivided into a physical method and a biochemical method.

Generally, physical preparation technologies of SNP mainly involve extrusion, grinding, and high-pressure homogenization. Wherein the extrusion method is a technology that feeds the raw materials of the prepared starch-water-crosslinker mixture into a high-temperature and high-pressure environment, then gelatinization and rupture occur after mechanical shearing by the screw [33]. Song et al. [34] used glycerol and glyoxal, respectively, as the plasticizer and crosslinking agents to prepare starch particles with an average particle size of 160 nm by adjusting the torque and rotation speed of the twin screw extruder. The results showed that the addition of crosslinking agents could significantly improve the screw shear force, resulting in starch particles with smaller particle sizes. However, during the extrusion process, the crystal structure was greatly damaged when the starch particle size was reduced, and the crosslinking agent was not food-grade. The milling method was also used to prepare SNPs. Ahmad et al. [35] milled water chestnut starch, lotus stem starch, and horse chestnut starch for 5 h using a planetary ball mill at 600 rpm to obtain nanoparticles. Gaining horse chestnut SNPs, water chestnut SNPs, and lotus root SNPs having average particle diameters of 343, 271, and 855 nm, respectively. Lin et al. [36] grounded potato raw starch by high-energy ball milling for 90 min to obtain spherical SNPs with an average particle size of about 120 nm, whose adsorption capacity was six times of raw starch.

Among biochemical methods, the acid hydrolysis method is the most widely used. As the crystalline lamellae of starch have strong acid resistance, the amorphous regions of starch will be acidolyzed preferentially. Therefore, the final hydrolysis product of nano-starch prepared by acidolyzation is starch nanocrystals (SNC) with high crystallinity. Jiang et al. [37] used waxy corn starch as the raw material, 3.16 mol/L concentrated sulfuric acid as the catalyst and reacted at 40 °C for 5 days to prepare sheet-like starch nanocrystals with a length of 80–100 nm and width of 30–60 nm. Since the reaction cycle of acid hydrolysis is extremely long, it is usually combined with ultrasound and enzymolysis. Agi et al. [38] dissolved cassava starch in low-concentration acetic acid and prepared plate-like starch nanoparticles with a size of 200 nm under the assistance of ultrasonic wave and enzymolysis. Experimental results showed that ultrasonic cavitation enhanced the combination of acetic acid molecules and the inside of large starch particles. It also accelerated the acidolysis rate in the crystallization zone. However, the auxiliary modification effect of ultrasonic technology was largely affected by equipment specifications and solvent system parameters, and large-scale production is difficult.

In general, the single “top-down” method is not enough to achieve satisfactory results, it needs to be combined with other methods. However, the synergistic use of methods still cannot solve defects such as complicated processes, long reaction cycles, and irregular product morphology completely. More importantly, due to the differences in specific production equipment and process parameters, the physicochemical properties of the products obtained by the “top-down” method mostly lack stability and are difficult to replicate.

The “bottom-up” method can be understood as a generalization of all SNPs prepared by the self-assembly approach, which refers to the transformation of native starch into the molecular state under certain conditions, and the re-nucleation of starch molecules to generate SNPs by adjusting the physical and chemical parameters of the system [25,39,40]. Specifically, the anti-solvent method [41], the recrystallization method [42], the polyelectrolyte complex method [43], the electrostatic spinning method [44], and the electrospray method [45]. Compared with the traditional “top-down” method, the product morphology synthesized by the “bottom-up” method is easy to regulate and control, the size and crystal structure are more uniform, and the product has higher stability in practical application.

The antisolvent method is an excellent “bottom-up” type nanoparticle preparation technology proposed by Fessi et al. [46] in 1989. It bases on the principle that raw materials are firstly dissolved in a good solvent and then rapidly mixed with a bad solvent to reduce the solubility of raw materials. The mixture is then nucleated and crystallized by inducing supersaturation of the solute and finally reconstituted into nanoscale particles [47,48]. Although the anti-solvent method is based on the precipitation effect of the solvent/anti-solvent system, the principle of the anti-solvent method determines that it can combine the properties of raw materials with the actual needs to differentiate into different production processes [49]. Such as interface deposition [50], solvent replacement [51], reverse precipitation [52], and micro emulsification [53], with great industrialization potential. The research by Chang Y. and Yang J. [54] showed that SNPs with a particle size of 150–300 nm and narrow size distribution could be prepared by reducing the molecular chain length of potato starch after treatment with β-amylase and then precipitating it by the anti-solvent method. The average particle size of 271.1 nm of SNPs was prepared by Yan et al. [55] and took anti-solvent precipitation and used debranched starch as the raw material. The content of resistant starch in SNPs was about 15.28% and the higher the volume of ethanol, the smaller the prepared SNPs particle size would be. The SNPs prepared by the anti-solvent method have the advantages of easy size adjustment, convenience, rapidness, environmental protection, low energy consumption, etc. with great development potential.

#### 2.2.2. Properties of Nano-Starch

Nano-starch has high biocompatibility and bioaccessibility, and its nano-scale size can promote the accurate delivery of active ingredients in the human body, which is conducive to improving bioavailability. It is an excellent bio-based carrier [56,57]. In recent years, many applications of SNP in the fields of food, medicine, and cosmetics are based on adsorption and embedding of targeted components. The starch-lutein nanoparticles prepared by anti-solvent precipitation technology exhibited a 75% reduction in the oxidation rate of lutein embedded in nano starch compared to that in the control group [58]. Acevedo-Guevara et al. [59] synthesized acetylated banana starch nanoparticles with an average particle size of about 250 nm as the carrier of curcumin. The results showed that the embedding rate of curcumin was improved by acetylation modification and the banana starch nanocarrier could induce the sustained release of curcumin in the stomach. The work of Alp et al. [60] has revealed that SNP can significantly reduce the release rate of CG-1527 and improve the delivery effect of histone deacetylase inhibitors in the treatment of breast cancer without interfering with its biological action mechanism. In the research by Yaseen et al. [61], spherical and polygonal nano-starch particles can inhibit the activity of α-amylase and theoretically achieve the purpose of slowing starch digestion and controlling blood glucose levels.

SNPs can also be used as fillers in composite materials to improve the mechanical strength and biodegradability of materials [62]. Dularia et al. [63] synthesized water chestnut SNPs by tape casting method and used them as the fillers of edible film. The results showed that the addition of SNP significantly increased the burst strength and thickness of the film, and reduced the water vapor transmission rate, solubility, and moisture content of the film. Roy et al. [64] compounded mung bean SNP prepared by acid hydrolysis with raw starch to prepare an edible composite film. Compared with the film prepared from raw starch, the composite film was more clear and more transparent, and the burst strength was increased from 868 g to 1265 g. Sharma et al. [65] prepared kidney bean SNP by acid hydrolysis and prepared the film by compounding it with raw starch. The results showed that the addition of SNP was conducive to reducing the moisture content and water vapor transmission rate and improving the packaging performance of the film.

## 3. Application of Starch Nanoparticle in Emulsion

### 3.1. Pickering Emulsifier Based on Starch Modification

**Figure 3:**
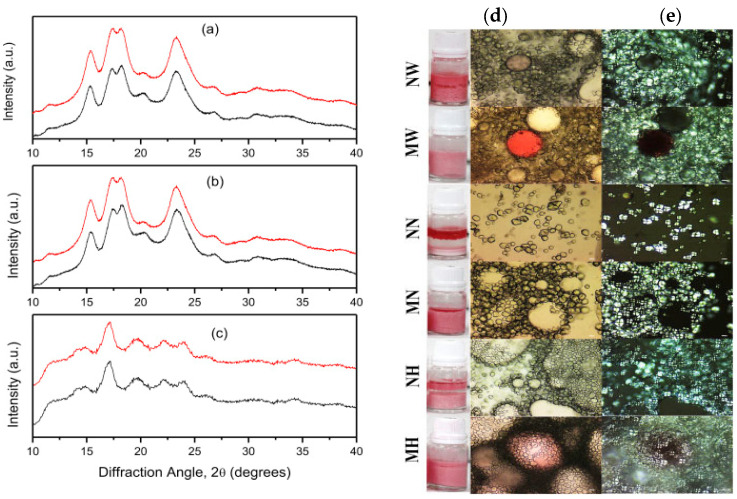
FTIR diffraction pattern of native (black line) and OSA-modified (red line) corn starches: (**a**) Waxy, (**b**) normal, and (**c**) Hylon VII. (For interpretation of the references to color in this figure legend, the reader is referred to the Web version of this article); Photomicrographs of transmitted light (**d**) and polarized light (**e**) microscopy of the emulsions stabilized with native corn starches and OSA (3%) modified starch at seven days. NW: native waxy, MW: Modified waxy, NN: native normal, MN: modified normal, NH: native Hylon VII, MH: modified Hylon VII. Conditions: 550 mg/mL oil, 0.2 mol/L NaCl. Scale bar 10 μm [66]. Reproduced with permission from Lopez-Silva, M.; Bello-Perez, L.A.; Agama-Acevedo, E.; Alvarez-Ramirez, J. Effect of amylose content in morphological, functional and emulsification properties of OSA modified corn starch; published by Elsevier, 2019.

The surface of raw starch contains a large number of hydroxyl, and it has a high degree of hydrophilicity and cannot be used as a stabilizer for Pickering emulsion, which requires hydrophobic modification. Emulsification tests implied that octenyl succinic starch ester was an effective emulsifier for the stabilization of oil-in-water emulsions. At present, the research on starch-based emulsifiers focuses on the modification of hydrophobic groups. The Octenyl succinic anhydride (OSA) modification is one of the most widely used hydrophobic modification strategies for food-grade starch, based on the principle of OSA grafting of starch molecules through esterification reaction under alkaline conditions [66] (Figure 3). However, the reaction conditions for the modification of OSA were rather harsh. On the one hand, the continuous addition of alkali was required as the prerequisite to ensure the reaction moved toward the esterification direction. On the other hand, the poor water solubility of OSA prevented it from fully entering the inside of starch particles, resulting in the distribution of OSA groups in starch is not uniform. The study showed that the degree of substitution of OSA starch was low, and the substitution efficiency was also unsatisfactory [67]. The OSA starch is the derivative that meets the need to stabilize emulsions. However, it is necessary to obtain modifications in OSA starch to increase its functions in emulsions. In addition, according to the regulations of the United States FDA, the degree of substitution of OSA starch cannot exceed 3%, which indicates that the application potential of OSA starch will be greatly limited by its safety [68].

### 3.2. Pickering Emulsifier Based on Starch Complex

**Figure 4:**
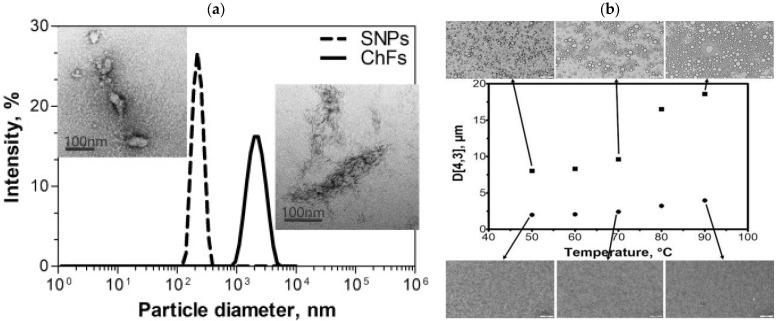
Size distribution of starch nanoparticles (dashed line) and chitin nanoparticles (solid line). The average size was 223.9 nm for starch and 1850 nm for chitin. TEM images show the morphologies of SNPs (left) and ChFs (right); scale bar = 100 nm (**a**); Droplet sizes of SNP/ChF-stabilized Pickering emulsions (C2S1) prepared with (●) and without (■) sonication at different temperatures. Inset: microscopic images of C2S1 emulsions. Scale bar inside panels = 50 μm (**b**) [69]. Reproduced with permission from Lee, Y.-S.; Tarté, R.; Acevedo, N.C. Synergistic effects of starch nanoparticles and chitin nanofibers on the stability of oil-in-water Pickering emulsions; published by Elsevier, 2021.

Due to the poor emulsifying ability of raw starch, starch, and another bio-based emulsifier can be combined to stabilize the emulsion through a synergistic effect [69] (Figure 4). Li et al. [70] compounded starch nanoparticles prepared by enzymolysis of pullulanase with cyclodextrin in a ratio of 1:2 to prepare a starch/cyclodextrin composite emulsifier, and found that the hydrophobic dispersion of cyclodextrin could significantly enhance the emulsification of starch. The emulsion still maintained a high emulsification index after six months of storage, showing excellent long-term stability. Guo et al. [71] synthesized a composite emulsifier of soluble starch and whey protein isolate and used it to prepare a high internal phase Pickering emulsion. The results showed that compared with the emulsion prepared from pure whey protein, the addition of starch significantly enhanced the emulsifying ability of whey protein and improved the retention rate of β-carotene. Shrestha et al. [72] prepared a soy protein isolate-banana-resistant starch conjugate and used it as a carrier of astaxanthin. The study found that compared with the emulsion stabilized by soy protein alone, the stability of the emulsion was enhanced by the presence of banana-resistant starch, which was conducive to the targeted release of astaxanthin.

Although the starch-based emulsifier can improve the stability of the emulsion under certain conditions, the combined emulsifier has great limitations. On the one hand, the emulsifying effect of the combined emulsifier easily fluctuates with the changes in the physical and chemical environment of the system. For example, the changes in temperature or pH result in the denaturation and precipitation of protein, and thus the loss of emulsifying ability [73,74]. On the other hand, emulsifiers such as protein and dextrin are expensive, and the production cost of the combined emulsifier is higher.

### 3.3. SNP-Based Pickering Emulsifier

**Figure 5:**
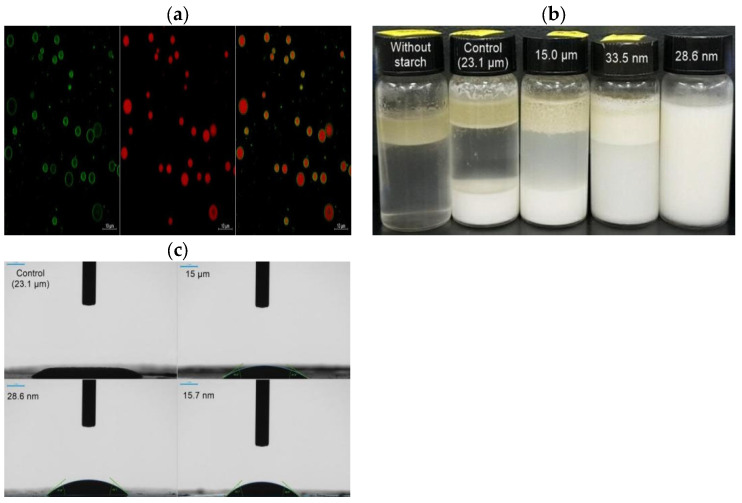
Dual-stained confocal laser scanning microscopic fluorescence image of o/w Pickering stabilized by starch nanoparticles with diameter of 28.6 nm. In this image, lipid (stained with oil red O) appears red and starch nanoparticle appears green (stained with oil blue O) (For interpretation of the references to color in this figure legend, the reader is referred to the web version of this article) (**a**); Change of emulsion morphology after storage for 24 h at an ambient temperature depending on mean particle sizes of starch sample and presence of starch (**b**); Contact angle of water droplet on a flat starch film made with different mean particle size of starch (**c**)) [77]. Reproduced with permission from Choi, H.-D.; Hong, J.S.; Pyo, S.M.; Ko, E.; Shin, H.-Y.; Kim, J.-Y. Starch nanoparticles produced via acidic dry heat treatment as a stabilizer for a Pickering emulsion: Influence of the physical properties of particles; published by Elsevier, 2020.

A study showed that reducing the size of starch particles was conducive to the formation of emulsion with smaller droplet sizes, and improved the storage stability of the emulsion [75]. Lu et al. [76] produced SNPs with a particle size of about 700 nm by medium milling and used them to prepare Pickering emulsion. The study found that although it was easy to agglomerate after milling, SNP still had good emulsion stability. Choi et al. [77] prepared SNPs with a particle size of about 30 nm by dry heat treatment of starch under acidic conditions (Figure 5). The study found that when the concentration of SNPs was higher than 9.1%, the emulsion index of SNPs-based Pickering emulsion was as high as 95%, and it had good freeze-thaw stability. Shao et al. [78] used taro starch as the raw material and prepared SNPs by the alkali soaking method. The study showed that the three-phase contact angle of SNPs with a particle size of about 460 nm was about 81.5°, and the emulsion prepared by SNPs had good emulsion stability, with the retention rate of embedded tea polyphenols as high as 67%.

A study showed that compared with proteins and lipids, the structural changes caused by heat-induced starch gelatinization might improve the stability of the emulsion [79]. Therefore, on the premise of not involving group modification and emulsifier component compounding, the raw starch nano-hierarchical structure was modified to prepare SNPs that can reflect the excellent nano-size effect in most application scenarios. The correlation between the nano-structure of SNPs and the emulsifying property was explored, which was conducive to expanding the application of starch-based emulsifiers.

## 4. Conclusions

Due to the small size of starch nanoparticles, it has high dispersibility and is easy to gelatinize during processing. It has the tendency of spontaneous agglomeration due to the high surface energy. Therefore, the size effect of starch nanoparticles varies in different environments, which directly affects their emulsifying ability. So far, there is still a lack of systematic discussion on the structure-activity relationship between the nano-structure of starch nanoparticles and their emulsion stability. Through the comprehensive investigation of the relationship between the nano-size effect of starch nanoparticles and their functional characteristics, as well as improving the performance by modifying starch nanoparticles and binding them with food-grade components such as fiber and protein, it is of great significance to give full play to the advantages of starch nanoparticles in practical applications.

## Data Availability

The data used to support the findings of this study can be made available by the corresponding author upon request.
